# Automated segmentation of the hypothalamus and associated subunits in brain MRI^☆^

**DOI:** 10.1016/j.neuroimage.2020.117287

**Published:** 2020-12

**Authors:** Benjamin Billot, Martina Bocchetta, Emily Todd, Adrian V. Dalca, Jonathan D. Rohrer, Juan Eugenio Iglesias

**Affiliations:** a Centre for Medical Image Computing, Department of Medical Physics and Biomedical Engineering, University College, London, UK; b Dementia Research Centre, Department of Neurodegenerative Disease, UCL Queen Square Institute of Neurology, University College, London, UK; c Martinos Center for Biomedical Imaging, Massachusetts General Hospital and Harvard Medical School, Boston, USA; d Computer Science and Artificial Intelligence Laboratory, Massachusetts Institute of Technology, Boston, USA

**Keywords:** Hypothalamus, Segmentation, Convolutional neural network, Public software

## Abstract

Despite the crucial role of the hypothalamus in the regulation of the human body, neuroimaging studies of this structure and its nuclei are scarce. Such scarcity partially stems from the lack of automated segmentation tools, since manual delineation suffers from scalability and reproducibility issues. Due to the small size of the hypothalamus and the lack of image contrast in its vicinity, automated segmentation is difficult and has been long neglected by widespread neuroimaging packages like FreeSurfer or FSL. Nonetheless, recent advances in deep machine learning are enabling us to tackle difficult segmentation problems with high accuracy. In this paper we present a fully automated tool based on a deep convolutional neural network, for the segmentation of the whole hypothalamus and its subregions from T1-weighted MRI scans. We use aggressive data augmentation in order to make the model robust to T1-weighted MR scans from a wide array of different sources, without any need for preprocessing. We rigorously assess the performance of the presented tool through extensive analyses, including: inter- and intra-rater variability experiments between human observers; comparison of our tool with manual segmentation; comparison with an automated method based on multi-atlas segmentation; assessment of robustness by quality control analysis of a larger, heterogeneous dataset (ADNI); and indirect evaluation with a volumetric study performed on ADNI. The presented model outperforms multi-atlas segmentation scores as well as inter-rater accuracy level, and approaches intra-rater precision. Our method does not require any preprocessing and runs in less than a second on a GPU, and approximately 10 seconds on a CPU. The source code as well as the trained model are publicly available at https://github.com/BBillot/hypothalamus_seg, and will also be distributed with FreeSurfer.

## Highlights

• A publicly available deep learning tool to segment the hypothalamus and its subunits.
• Our tool outperforms inter-rater accuracy and approaches intra-rater precision level.
• It can robustly generalise to unseen heterogeneous datasets.
• It yields a rejection rate of less than 1% in a QC analysis performed on 675 scans.
• It detects subtle subunit-specific hypothalamic atrophy in Alzheimer’s Disease.

## 1 Introduction

### 1.1 Motivation

The hypothalamus is a cerebral structure, that is part of the diencephalon, and located below the thalamus. The hypothalamus plays a central role in controlling many vital functions, including food intake and perception of satiety (Minokoshi, Alquier, Furukawa, Kim, Lee, Xue, Mu, Foufelle, Ferré, Birnbaum, Stuck, Kahn, 2004, Saper, Chou, Elmquist, 2002), circadian rhythms (i.e., sleep-wake pattern) (Saper et al., 2005), immune and endocrine response (Clarke, 2015, Cross, Markesbery, Brooks, Roszman, 1980, Luiten, ter Horst, Steffens, 1987), thermoregulation (Boulant, 1981), and cardiovascular activity (Rahmouni, 2016). The hypothalamus is subdivided into approximately a dozen nuclei (depending on subdivision criteria), each with different functions and specialised cell groups (Saper, 1990). Because of its many functions, the hypothalamus is affected by a large number of disorders, such as eating and sleep disorders (Mignot, Taheri, Nishino, 2002, Warren, Voussoughian, Geer, Hyle, Adberg, Ramos, 1999), Alzheimer’s Disease (AD) (Ishii and Iadecola, 2015), Parkinson’s Disease (Langston, Forno, 1978, Politis, Piccini, Pavese, Koh, Brooks, 2008), and frontotemporal dementia (Ahmed, Latheef, Bartley, Irish, Halliday, Kiernan, Hodges, Piguet, 2015, Piguet, Petersén, Yin Ka Lam, Gabery, Murphy, Hodges, Halliday, 2011). These disorders affect the hypothalamic subnuclei differently, and often alter only a subset of them (Bocchetta, Gordon, Manning, Barnes, Cash, Espak, Thomas, Modat, Rossor, Warren, Ourselin, Frisoni, Rohrer, 2015, Goudsmit, Hofman, Fliers, Swaab, 1990). Therefore, the ability to study hypothalamic nuclei individually *in vivo* is of paramount importance for a better understanding of these disorders.

Due to its superior soft tissue contrast, magnetic resonance imaging (MRI) is the technique of choice for studying the human brain *in vivo*, including the hypothalamus (Baroncini et al., 2012). A prerequisite for most quantitative analyses of hypothalamic substructures in MRI scans is the delineation of their contours, a task known as image segmentation. The resulting labelled images can then be used for an array of subsequent tasks such as *in vivo* volumetry, morphology, and connectivity analyses (Bocchetta, Gordon, Manning, Barnes, Cash, Espak, Thomas, Modat, Rossor, Warren, Ourselin, Frisoni, Rohrer, 2015, Makris, Swaab, van der Kouwe, Abbs, Boriel, Handa, Tobet, Goldstein, 2013). Different protocols have been proposed to manually segment the hypothalamic subunits in brain MRI scans (Baroncini, Jissendi, Balland, Besson, Pruvo, Francke, Dewailly, Blond, Prevot, 2012, Bocchetta, Gordon, Manning, Barnes, Cash, Espak, Thomas, Modat, Rossor, Warren, Ourselin, Frisoni, Rohrer, 2015, Makris, Swaab, van der Kouwe, Abbs, Boriel, Handa, Tobet, Goldstein, 2013). Although manual segmentation is still considered the gold standard in terms of accuracy, it remains a time-consuming and tedious procedure (e.g. delineation of the hypothalamic subunits typically requires 2 to –3 hours per scan at 1 mm resolution for an expert tracer), and thus is not scalable to large datasets. Moreover, hypothalamic substructures are difficult to delineate on MR scans, making the segmentations hardly reproducible and severely prone to inter- and intra-rater variability.

Automated algorithms have been introduced to tackle these problems, as they do not require human intervention and enable reproducible segmentations of large datasets. However, very few automated strategies have been proposed to segment the whole hypothalamus in structural MRI scans (D’Haese, Duay, Merchant, Macq, Dawant, 2003, Orbes-Arteaga, Cárdenas-Peña, Álvarez, Orozco, Castellanos-Dominguez, 2015, Rodrigues, Rezende, Zanesco, Hernandez, Franca, Rittner, 2020, Thomas, Beyer, Lewe, Zhang, Schindler, Schönknecht, Stumvoll, Villringer, Witte, 2019), and no automated method exists – to the best of our knowledge – for hypothalamic nuclei segmentation.

### 1.2 Related work

Brain MRI segmentation has traditionally been dominated by atlas based techniques. The simplest way to automatically segment an MRI scan is to use a single atlas. In this technique, a labelled training 3D image (i.e., an atlas) is registered to a test scan, whose final segmentation is obtained by applying the resulting transformation to the labels (Collins, Holmes, Peters, Evans, 1995, Iosifescu, Shenton, Warfield, Kikinis, Dengler, Jolesz, McCarley, 1997). This strategy is straightforward but its accuracy highly depends on the quality of the registration, which in turns depends on the similarity between the two scans. Moreover, its accuracy is especially limited in small structures such as the hypothalamus (D’Haese et al., 2003), and the choice of atlas introduces a bias in the results.

An alternative strategy is to use multiple labelled scans in order to increase the anatomical variability covered by the model, while limiting the bias introduced by the use of a single atlas. Two techniques emerged from this principle. The first one, called multi-atlas segmentation (MAS), consists in individually registering all the available atlases onto the test scan, and applying the warps to their corresponding label maps. All the warped labels are then merged into one final segmentation with a label-fusion algorithm (Artaechevarria, Munoz-Barrutia, Ortiz-de Solorzano, 2009, Heckemann, Hajnal, Aljabar, Rueckert, Hammers, 2006, Iglesias, Sabuncu, 2015, Isgum, Staring, Rutten, Prokop, Viergever, van Ginneken, 2009, Sabuncu, Thomas Yeo, Van Leemput, Fischl, Golland, 2010). Recent studies have shown that MAS yields relatively accurate results for the whole hypothalamus (Orbes-Arteaga, Cárdenas-Peña, Álvarez, Orozco, Castellanos-Dominguez, 2015, Thomas, Beyer, Lewe, Zhang, Schindler, Schönknecht, Stumvoll, Villringer, Witte, 2019). However, this strategy is computationally expensive due to the high number of required registrations, although the running time can now be considerably decreased with deep learning based registration methods (Dalca, Balakrishnan, Guttag, Sabuncu, 2019, de Vos, Berendsen, Viergever, Sokooti, Staring, Išgum, 2019).

The second technique is Bayesian segmentation, in which all training atlases are summarised into a single probabilistic atlas, that is combined with deformation (prior) and image intensity (likelihood) models to form a generative model. Segmentation is obtained by “inverting” this generative model using Bayesian inference. This second strategy is adaptive to MRI contrasts when unsupervised likelihood models are used (Ashburner, Friston, 2005, Puonti, Iglesias, Van Leemput, 2016), and faster than traditional MAS. For these reasons, Bayesian segmentation remains used by all major neuroimaging packages (FreeSurfer (Fischl, 2012), SPM (Ashburner and Friston, 2005), FSL (Patenaude et al., 2011)). Nevertheless, none of these packages segment the hypothalamus nor its subregions. For example, in FreeSurfer they are directly encompassed in a broader region called “ventral DC”, including numerous other small structures.

Modern automated image segmentation techniques rely on deep neural networks (Kamnitsas et al., 2017), which have been recently applied to segment the whole hypothalamus (as opposed to hypothalamic subunits) with relatively high accuracy (Rodrigues et al., 2020). By learning a set of convolutional kernels, convolutional neural networks (CNN) can effectively capture highly non-linear distributions between inputs and outputs. U-net architectures, which extract discriminative features at progressive levels of resolution, now represent the state-of-the-art class of methods in terms of segmentation accuracy (Ronneberger et al., 2015). The success of deep learning networks has also been reinforced by the extremely short processing times, enabling to retrieve segmentations in seconds (Akkus, Galimzianova, Hoogi, Rubin, Erickson, 2017, Dou, Chen, Yu, Zhao, Qin, Wang, Mok, Shi, Heng, 2016).

However, deep learning methods traditionally require numerous training pairs of images and associated ground truth to prevent overfitting. In the case of brain MRI segmentation, insufficient training data leads to models that are sensitive to changes in image resolution and MR contrast (Akkus et al., 2017); this is one of the reasons why Bayesian methods are still preferred by the neuroimaging packages mentioned above. This problem is now being ameliorated with data augmentation techniques, which enable to artificially increase the size of the training dataset by modifying its intensity distributions or spatially deforming its shape in a random manner. This strategy greatly reduces overfitting even when a small number of training examples is used (Jog and Fischl, 2018; Zhao et al., 2019). Moreover, there is converging evidence that performing data augmentation beyond realistic shape and appearance helps networks to better generalise on previously unseen data at test time (Billot, Greve, Van Leemput, Fischl, Iglesias, Dalca, Chaitanya, Karani, Baumgartner, Becker, Donati, Konukoglu, 2019, Eaton-Rosen, Bragman, Ourselin, Cardoso, 2018, Zhao, Balakrishnan, Durand, Guttag, Dalca, 2019).

### 1.3 Contribution

The central contribution of this work is to present the first fully automated tool to segment the hypothalamus and its internal subunits on MRI scans. The presented framework requires no preprocessing and relies on a state-of-the-art deep neural network, trained with a dataset of 37 T1-weighted brain MRI scans with corresponding manual delineations. Using Dice scores and surface distances, we first demonstrate that our method is able to accurately segment the hypothalamic subunits, outperforming the well-established MAS framework as well as human inter-rater reliability scores, and approaching intra-rater levels. Using the same metrics, we then show that our framework is able to sustain this performance level on a separate, heterogeneous, labelled dataset with four subjects. We further validate the robustness of the presented network with two experiments on 675 MRI scans from the heterogeneous ADNI dataset. ADNI includes scans that were acquired with different scanners, sequences, and parameters, and often display AD-related pathology. Despite these differences, visual quality control (QC) performed by an expert rater (blindly of the subjects age, gender or condition) reveals that reliable segmentations are obtained in more than 99% of the cases, demonstrating the robustness of our approach. Moreover, our model successfully detects subtle, subunit-specific hypothalamic atrophy in AD on this dataset. The code and the weights of the trained network are publicly available at: https://github.com/BBillot/hypothalamus_seg, and will be distributed with FreeSurfer.

## 2 Methods

### 2.1 Data augmentation

**Fig. 1:**
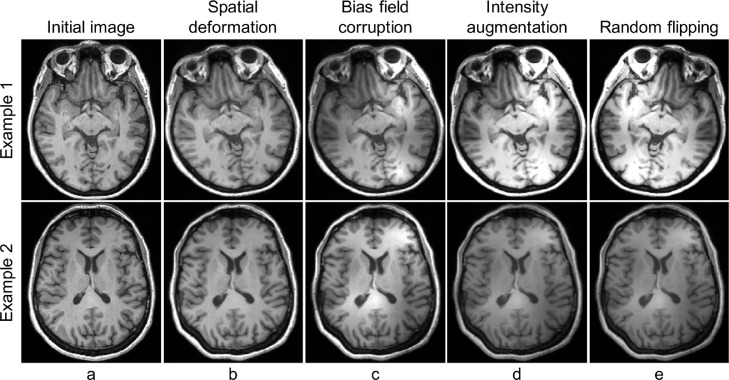
Axial slices of intermediate image volumes obtained at different steps of the proposed augmentation model. First, the input image (a) is spatially deformed (b). We then apply a random bias field (c), and further global intensity augmentation (d). Finally the image is flipped along the right/left axis with a probability of 0.5. Each row corresponds to a different subject. The displayed slices correspond to the same coordinate in the inferior-superior axis. Augmentation is performed on the fly, and all random parameters are resampled at every step in training, such that the network is never exposed to the same image twice.

We achieve segmentation of the whole hypothalamus and its subregions by training a 3D convolutional neural network on manually labelled T1-weighted scans. Training pairs of 3D MRI scans (also referred to as images in this manuscript) and segmentations are first considerably augmented in order to completely avoid preprocessing at test time. Augmentation makes our network resilient against expected variations in subject positioning, imaging artefacts (noise, bias field), and contrast variations in T1-weighted brain MRI scans due to differences in magnet strength, pulse sequence, and acquisition hardware. Training pairs are randomly augmented on the fly (i.e. directly during training), such that the network is never exposed twice to the same image. The different steps of the augmentation model are detailed below, and illustrated in Fig. 1.

Our augmentation model starts by defining a diffeomorphic non-linear transformation for elastic image deformation. This is achieved by randomly sampling a small-size 3D vector field (e.g., 10 × 10 × 10 × 3) with Gaussian noise, linearly interpolating it to full image size to obtain a smooth stationary velocity field, and integrating the result (Arsigny et al., 2006). Using diffeomorphic transforms parametrised by stationary velocity fields ensures that the deformations are invertible, so that they produce neither holes nor foldings.

The image subsequently undergoes an affine transformation encoded by a 4 × 4 matrix in homogeneous coordinates. This matrix can be decomposed into the product of six matrices: three rotations (one around each axis), along with scaling, shearing and translation matrices. All these transformations are parametrised by coefficients randomly sampled from uniform distributions of predefined ranges. In practice, images are resampled with linear rather than nearest neighbour interpolation, which would introduce high frequency noise and strongly hinder anatomical coherence. However, because linear interpolation tends to smooth intensities, the elastic and linear transforms are applied simultaneously to avoid unnecessary resampling steps, and thus excessive smoothing (Fig. 1(b)).

The augmentation model also accounts for non-uniformities in the magnetic field commonly observed in MR scanners (Simmons et al., 1994). Because this phenomenon translates into intensity inhomogeneities smoothly varying across MRI scans (Sled and Pike, 1998), we model it with a multiplicative smooth field. As before, we sample a small low resolution field (e.g., of size 4 × 4 × 4), and upscale it to image size with linear interpolation. Then, we take the voxel-wise exponential to ensure the non-negativity of this field. Finally, we multiply the spatially deformed scan by the obtained bias field to corrupt its intensities (Fig. 1(c)).

In order to make the network robust against acquisition procedures, we add further global intensity augmentation by shifting the brightness and contrast of the image with randomly sampled values (Fig. 1(d)). The obtained scan is subsequently flipped along the right-left axis with a probability of 0.5 (Fig. 1(e)), and randomly cropped to a size of 160^3^, which is more than large enough to ensure that the hypothalamus is always present in the resulting scan. Finally, intensities are rescaled between [0,1] with min-max normalisation. Additional examples of augmented images are shown in the Supplementary materials (Fig. S1).

Finally, as we aim to produce paired images and segmentations, the manual delineations of hypothalamic subunits undergo the same spatial transformations as their corresponding image, and left and right labels are swapped when lateral flipping occurs. The spatial transformation occurs in one-hot encoding space, i.e., we deform a binary map for each label with linear interpolation, yielding deformed soft segmentations. This strategy avoids the high-frequency noise introduced by direct deformation of discrete labels with nearest neighbour interpolation.

### 2.2 Network architecture and learning

**Fig. 2:**
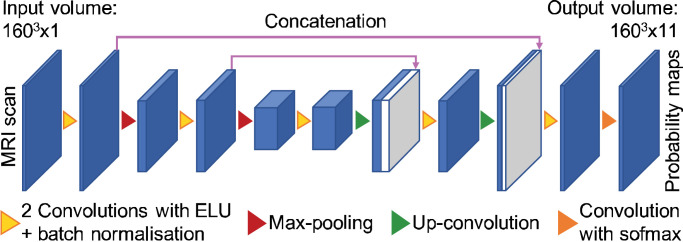
Architecture of the 3D deep learning network. The first layer comprises 24 kernels, this number being doubled after each max-pooling, and halved after each up-convolution.

Segmentation of the hypothalamic subunits is achieved by training a CNN on the outputs of the augmentation model. The architecture of the network, based on the state-of-the-art 3D U-net model (Ronneberger et al., 2015), is represented in Fig. 2. It begins with a contracting path, which extracts discriminative information from consecutively downsampled feature maps. The prediction of the network is then progressively build at increasing resolution levels along an expanding path. The specificity of the U-net architecture relies on concatenating feature maps of corresponding resolution between the two paths. By incorporating information of lower abstraction level, these links provide context to the expanding path, thus enabling a more accurate prediction of the output (Ronneberger et al., 2015).

The architecture hyperparameters are chosen as explained in the Experiments and results section. The network consists of three resolution levels, where a level refers to all feature maps between two max-pooling (downsampling) or upsampling operations. Each convolution is performed with kernels of size 3 × 3 × 3. The first convolution includes 24 kernels, this number being doubled after each max-pooling, and halved after each up-convolution. All layers, except the last, use the Exponential Linear Unit (ELU) activation function, as they yield improved learning characteristics compared to previously used ReLU, LReLU or PReLU operations (Clevert et al., 2015). The last layer has a softmax activation function, which enables to obtain an output under the form of a differentiable probabilistic map for each label. The loss function is computed by calculating the average of the soft Dice coefficients between the predicted probability maps and the ground truth soft label maps (Milletari et al., 2016). Dice coefficients measure the spatial overlap of two segmentations and ranges from 0 (no overlap) to 1 (perfect overlap). As opposed to other metrics such as the cross-entropy, using the average Dice score uniformly integrates information from all labels, regardless of their volume. If $X=\{x_{i}\}$and $Y=\{y_{i}\}$respectively represent the predicted and ground truth probability maps for a given label, their soft Dice coefficient (SDC) is given by:

(1) $$SDC\left(X,Y\right)=2\times \frac{\sum_{i}x_{i}y_{i}}{\sum_{i}x_{i}^{2}+\sum_{i}y_{i}^{2}}.$$

Optimising the Dice score from randomly initialised weights is problematic due to the low gradient of the Dice loss function far away from any maximum. We mitigate this problem by pre-training the model with a sum of squared differences loss function (SSD) on the output of the penultimate layer, i.e., the activations that are used as input to the softmax. Specifically, we teach this truncated version of the original network to output at each voxel an arbitrary value of *T_target_* for the correct label ($y_{i}=1$), and $-T_{target}$for the incorrect ones ($y_{i}=0$):

(2) $$SSD\left(X,Y\right)=\sum_{i}{\left[x_{i}-T_{target}\left(2y_{i}-1\right)\right]}^{2}.$$

This loss has a much stronger gradient than the Dice score away from its maxima, and by running it for a predefined number of iterations, we bring the network weights to a more favourable region of the optimisation landscape. We can then resume the training with the Dice loss, which enables to obtain precise and robust segmentations for small structures during inference, as explained above. In addition to this pre-training, we use batch normalisation, which has been shown to accelerate the training of deep learning networks by normalising the outputs of each layer and thus reducing the internal covariance shift (Ioffe and Szegedy, 2015).

### 2.3 Manual segmentation

**Fig. 3:**
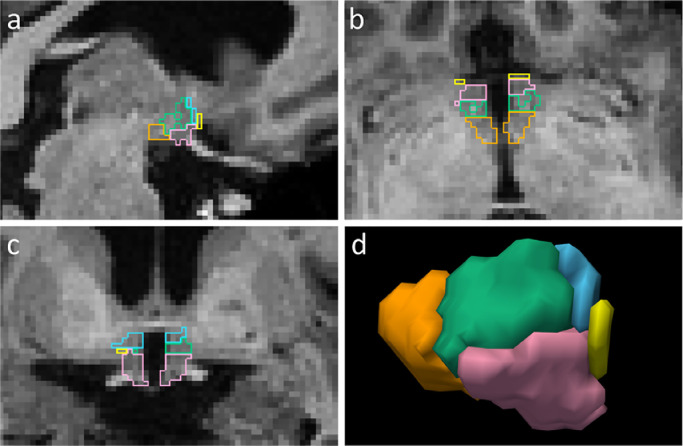
Example of manually segmented hypothalamus in (a) sagittal, (b) axial and (c) coronal views. (d) 3D rendering of the right hypothalamus. Subunits are depicted in different colours: a-sHyp in blue, a-iHyp in yellow, supTub in green, infTub in pink, and posHyp in orange.

The subdivision of the whole hypothalamus into subunits follows the protocol introduced by Makris et al. (2013). Considering the small size of the hypothalamic nuclei, this method uses visible anatomical landmarks to regroup them into five subunits, which can be reliably segmented at standard 1 mm resolution: (*i*) the anterior-superior hypothalamus (a-sHyp); (*ii*) the anterior-inferior hypothalamus (a-iHyp); (*iii*) the superior tuberal hypothalamus (supTub); (*iv*) the inferior tuberal hypothalamus (infTub); and (*v*) the posterior hypothalamus (posHyp). The composition of each subunit is detailed in Table 1, and an example of manual delineation is illustrated in Fig. 3.

**Table 1:** Grouping of the hypothalamic nuclei into subunits, according to Bocchetta et al. (2015); Makris et al. (2013).

Subunit	Nuclei included
Anterior-superior (a-sHyp)	preoptic area; paraventricular nucleus (PVN)
Anterior-inferior (a-iHyp)	suprachiasmatic nucleus; supraoptic nucleus (SON)
Superior tubular (supTub)	dorsomedial nucleus; PVN; lateral hypothalamus
Inferior tubular (infTub)	infundibular (or arcuate) nucleus; ventromedial nucleus; SON; lateral tubular nucleus; tuberomamillary nucleus (TMN)
Posterior (posHyp)	mamillary body (including medial and lateral mamillary nuclei); lateral hypothalamus; TMN

## 3 Experiments and results

In this section, we present four sets of experiments aiming to validate the proposed method. We first assess the reliability of manual subunit segmentation with an inter- and intra-rater reproducibility study. In the second experiment, we train the network and compare the accuracy of its automated segmentations against the reliability scores of the first experiment and MAS. In the third experiment, we test the robustness of the automated method against differences in acquisition by evaluating it on a small heterogeneous labelled dataset, and by performing a QC analysis on the segmentations of a large sample of subjects from the heterogeneous, publicly available ADNI dataset. In the fourth and final experiment, we assess the ability of our method to detect atrophy patterns related to AD, also using ADNI. This fourth setup is representative of the type of application our method is designed for.

### 3.1 MRI data

The first two experiments employ whole head scans from a dataset of 37 subjects (referred to as “internal dataset”) described in Bocchetta et al. (2015). We randomly divide this dataset between training, validation and testing subsets comprising 13, 6, and 18 subjects, respectively. All scans are unprocessed T1-weighted MP-RAGE 3D images at isotropic 1.1 mm resolution with size 256 × 256 × 208. They were all acquired on a 3T Siemens scanner, with parameters: TR =2200 ms, TI =900 ms, TE =2.9 ms, $\alpha =10^{\circ }$. Subjects are equally divided between healthy controls and subjects fulfilling the criteria for the diagnosis of behavioural variant frontotemporal dementia (FTD). The control subjects are 56.4 ± 14.3 years old, whereas the FTD group is 63.3 ± 9.1 years old.

These 37 images were manually segmented following the protocol described in the Methods section. The produced segmentations consist of eleven labels: one for the background and five for the subunits of each hypothalamus (right and left). Delineation of this dataset is performed with the help of corresponding T2-weighted scans. These are acquired using a fast spin echo/SPACE sequence with following parameters: TR =3200 ms, apparent TE =105 ms and variable refocusing pulse flip angle to achieve T2-weighting. Both T1-weighted and T2-weighted scans are acquired during the same session, and are of same size and resolution. Neither the T1 nor the T2 scans were preprocessed in any way.

We use two other datasets to evaluate the robustness of our method. The first one (referred to as “external dataset”) contains four subjects: two from the HCP dataset (Sotiropoulos et al., 2013), and two from the lower-quality, 1.5T, IXI dataset (IXI, 2015). T1 and T2-weighted whole head scans are available for all four subjects, which enables us to apply the previously described protocol to manually segment the hypothalamic subunits. The HCP data is resampled from isotropic 0.7 mm native resolution to 1 mm, whereas the IXI scans are directly available at isotropic 1 mm resolution. Additional information on the acquisition can be found in IXI (2015); Sotiropoulos et al. (2013). Other than downsampling the HCP scans, no preprocessing steps are performed.

The second evaluation dataset is a subset of 675 subjects from the ADNI dataset. All scans are T1 weighted and acquired at approximately 1 mm isotropic resolution. The scans are acquired on a wide array of different scanners with varying parameters and sequences; further details on the acquisition can be found on the ADNI website (http://adni-info.org). All subjects are tested for cognitive impairment and AD with the Alzheimer’s disease Assessment Scale test (ADAS). The population includes 183 elderly control subjects (94 males, 89 females), 358 subjects with different stages of mild cognitive impairment (MCI; 182 males, 176 females), and 134 subjects with AD (73 males and 61 females). Subjects are within the same range of ages: 75.3 ± 8.2 years for males against 72.6 ± 7.8 for females; 72.9 ± 9.4 years for controls against 75.4 ± 9.1. for MCI, and 76.0 ± 7.2. for AD. No preprocessing was performed on these images. Ground truth segmentations are not available for this dataset.

The ADNI was launched in 2003 by the National Institute on Ageing, the National Institute of Biomedical Imaging and Bioengineering, the Food and Drug Administration, private pharmaceutical companies and non-profit organisations, as a $60 million, 5-year public-private partnership. The main goal of ADNI is to test whether MRI, positron emission tomography (PET), other biological markers, and clinical and neuropsychological assessment can be combined to analyse the progression of mild cognitive impairment (MCI) and early AD. Markers of early AD progression can aid to develop new treatments and monitor their effectiveness, as well as decrease the time and cost of clinical trials. The Principal Investigator of this initiative is Michael W. Weiner, MD, VA Medical Center and University of California – San Francisco. ADNI has been followed by ADNI-GO and ADNI-2. These three protocols have recruited over 1,500 adults (ages 55–90) from over 50 sites across the U.S. and Canada to participate in the study, consisting of cognitively normal older individuals, people with early or late MCI, and people with early AD. Subjects originally recruited for ADNI-1 and ADNI-GO had the option to be followed in ADNI-2.

### 3.2 Evaluation metrics

Similarity between predicted and ground truth segmentations is first assessed by computing Dice coefficients for the whole hypothalamus and each hypothalamic subunit. Prior to evaluation, the soft predicted label maps are converted to categorical encoding by keeping the most probable label at each voxel. Therefore, instead of computing soft Dice scores defined in (1), we now use hard Dice coefficients to evaluate the accuracy of categorical segmentations, as in common practice in neuroimaging. We emphasise that hard Dice cannot be used in training, since it is not a differentiable function. If *X* and *Y* are corresponding structures in two different segmentations, their (hard) Dice score is given by:

(3) $$Dice\left(X,Y\right)=2\times \frac{∥X\cap Y∥}{∥X∥+∥Y∥}.$$

However, Dice scores are very sensitive to small spatial shifts when comparing small and thin structures, such as the hypothalamic subunits. Therefore, we also report here the average boundary distance (*d_A_*) and the Hausdorff distance (*d_H_*), which respectively measure the average and the maximum distance between the surfaces of two segmentations. If *X* and *Y* are corresponding structures in two different segmentations, with surfaces *S_X_* and *S_Y_*, these two metrics are given by:

(4) $$d_{A}\left(X,Y\right)=\mathrm{mean}\left\{\underset{x\in S_{X}}{\mathrm{mean}}\underset{y\in S_{Y}}{\inf}d\left(x,y\right),\underset{y\in S_{Y}}{\mathrm{mean}}\underset{x\in S_{X}}{\inf}d\left(x,y\right)\right\},$$

(5) $$d_{H}\left(X,Y\right)=\max\left\{\underset{x\in S_{X}}{\sup}\underset{y\in S_{Y}}{\inf}d\left(x,y\right),\underset{y\in S_{Y}}{\sup}\underset{x\in S_{X}}{\inf}d\left(x,y\right)\right\},$$

where ‖ · ‖ represents cardinality and *d* is the euclidean distance. As distances, *d_A_* and *d_H_* are both sought to be minimised (they are equal to zero in case of a perfect segmentation). Because they depend on the surface rather than the size, *d_A_* and *d_H_* are less biased for small structures than Dice coefficients. These two metrics are complementary: the average boundary distance gives a good representation of spatial alignment, whereas the Hausdorff distance evaluates the robustness of a segmentation, as it is determined by the furthest misclassified voxel.

### 3.3 Experiments

#### 3.3.1 Intra and inter-rater reproducibility study

Because of the general lack of contrast in hypothalamic region for both T1-weighted and T2-weighted scans, drawing the contours of the hypothalamus and its subunits is a challenging task. As manual delineation is considered the gold standard in segmentation, we first assess its reliability in order to put the results of our automated framework into context. With this purpose, we conduct an extensive inter- intra-rater variability experiment using the protocol described in the Section 2.

This experiment starts by randomly drawing ten subjects from the 17 test scans of our internal dataset. These selected subjects were relabelled by two raters: once by an expert rater, who already segmented the whole internal dataset for a previous publication (Bocchetta et al., 2015); and once by a trainee rater, who was trained for this task by the expert rater. All segmentations are considered to be independent, as four years elapsed between the two sets of delineations made by the expert rater, and because the second rater was trained on a different set of scans not included in this analysis. The intra-rater variability study is performed by measuring the similarity between the two sets of segmentations made by the expert rater, whereas the inter-rater study compares both delineations from the expert rater with the ones of the trainee rater.

Table 2 reports the average similarity scores obtained for the whole hypothalamus and subunits on the ten subjects considered in this experiment. The inter-rater scores are all worse than the intra-rater ones (Dice score difference =0.15±0.02,average boundary distance difference =0.24mm  ± 0.03, Hausdorff distance difference =0.51mm  ± 0.13). The intra-rater variability is significantly lower (*p*  < 0.05) for all structures and metrics, according to paired, non-parametric tests (Wilcoxon signed-rank).

**Table 2:** Inter/intra-rater reproducibility scores for manual segmentation of the whole hypothalamus and all subunits. Stars indicate the level of statistical significance (one-sided Wilcoxon, non-parametric signed-rank test) between intra-rater and inter-rater results (* *p* < 0.05, ** *p* < 0.01).

	Type	whole	a-sHyp	a-iHyp	supTub	infTub	posHyp
Volume (mm^3^)		649.5	23.1	16.3	94.9	103.7	86.8
Dice coefficient	intra	0.89**	0.70*	0.54*	0.82**	0.87**	0.87**
	inter	0.75	0.55	0.42	0.67	0.72	0.67
Average distance (mm)	intra	0.23**	0.33*	0.58*	0.24**	0.20**	0.22**
	inter	0.49	0.53	0.83	0.46	0.41	0.51
Hausdorff distance (mm)	intra	1.91**	1.64*	2.28*	1.70**	1.54**	1.51*
	inter	2.92	2.03	2.97	2.35	1.91	1.96

The whole hypothalamus yields very good intra-rater scores for the Dice coefficient and average boundary distance (respectively 0.89 and 0.23 mm). The results are more moderate in the inter-rater case (Dice =0.75,$d_{A}=0.49$mm), but remain at a good level considering the small size of the hypothalamus. In both cases, these results are similar to the best scores achieved by individual nuclei. The inverse trend is observed for the Hausdorff distance, where the whole hypothalamus yields scores comparable to the worst subunit.

By comparing the overall results of all subregions, we observe that the best reliability scores are achieved for the posterior and tubular subunits. The intra-rater study yields its best results for the posHyp and infTub regions, while the inter-rater achieves its best scores for the infTub subunit. On the contrary, the anterior nuclei yield noticeably inferior scores, with the a-iHyp unit achieving the lowest scores for both intra-rater and intra-rater experiments. A similar pattern is observed through statistical tests, where the difference between intra and inter-rater scores is slightly less significant for the anterior subunits: 0.01 < *p* < 0.05 for anterior nuclei, and *p* < 0.01 for posterior and tubular nuclei (except for the Hausdorff distance for the posHyp region, where p=0.013).

#### 3.3.2 Automated segmentation

In this section we explain how we train the proposed automated framework and evaluate its accuracy. The network is trained on the 13 training subjects of out internal dataset, and we use the validation subset to tune the architecture and hyperparameters without bias, by selecting the model with the lowest validation loss at the end of training (i.e., highest average soft Dice). The validation curve for the winning architecture described in the Methods section (which was *not* inspected during training) clearly shows that there was no overfitting (see Figures S2 and S3). Such architecture yields the best loss among several combinations of the following parameters: number of resolution levels (2, 3, 4, 5, 6), number of layers per level (2, 3), size of the convolution kernels (3 × 3 × 3, 5 × 5 × 5), number of features for each convolution (constant throughout the network, or doubled after each max-pooling and halved after each up-sampling), and activation function (ELU, RELU, leaky RELU). We also consider using dropout layers with different probabilities (from 0.1 to 0.5 by increments of 0.1), but these are abandoned due to substantial decrease in performance (Dice scores lower by at least 15%). Finally, after investigating the effect of different values for the learning rate ($10^{-3},$$10^{-4},$$10^{-5}$), and the learning rate decay (0, $10^{-1},$$10^{-2}$), these are set to $10^{-4}$and $10^{-2},$respectively.

Networks are trained with an ADAM optimiser (Kingma and Ba, 2017) for 100,000 steps, which is enough for the loss function to converge in all cases (e.g., as in Fig. S2), and which takes around 80 hours on an Nvidia Titan Xp GPU. The weighted sum of squares loss is used for the first 5,000 steps as a pre-training phase, and is then replaced by the average Dice coefficients loss (Eq. (1)) for the remaining steps. The batch size is set to 1 due to limitations in GPU memory, but this is balanced by the fact that the loss function and gradient are estimated on a high number of voxels (i.e. 160^3^). In order to measure the effectiveness of the data augmentation, an additional model was trained without performing any of the augmentation steps (except for the random cropping). However, we do not report these results here as this model produces segmentations of extremely poor quality (average Dice score below 0.1 for the whole hypothalamus). Our model is implemented in Keras (Chollet, 2016) with a Tensorflow (Abadi et al., 2016) backend, and relies on the Neuron (Dalca et al., 2018) ans Lab2Im (Billot et al., 2020) python packages.

The quality of the results is assessed by computing the same similarity metrics as before (i.e. Dice coefficient, average boundary distance, and Hausdorff distance), between predictions and corresponding manual delineations. The network is trained five times to reduce the fluctuations caused by the stochastic processes occurring during training (example selection, data augmentation, weights initialisation). The similarity scores for a given test subject are obtained by: running the corresponding T1-weighted scan with the five networks, computing the scores for each of the five predictions, and averaging the results of each model.

We compare the segmentations of the proposed network to results obtained with a MAS approach. MAS is a natural competing method for our framework, since it is a well established strategy for automated segmentation in neuroimaging (Artaechevarria, Munoz-Barrutia, Ortiz-de Solorzano, 2009, Heckemann, Hajnal, Aljabar, Rueckert, Hammers, 2006, Sabuncu, Thomas Yeo, Van Leemput, Fischl, Golland, 2010), and has recently been applied to segment the whole hypothalamus with relatively high accuracy (Orbes-Arteaga, Cárdenas-Peña, Álvarez, Orozco, Castellanos-Dominguez, 2015, Thomas, Beyer, Lewe, Zhang, Schindler, Schönknecht, Stumvoll, Villringer, Witte, 2019). In order for the results to be comparable, the division between training, validation and testing subsets is kept the same as for the network. Segmentations are computed by: (*i*) registering all training scans to the test scan with NiftyReg (Modat et al., 2010), using default parameters; (*ii*) applying the obtained deformations to the training delineations; and (*iii*) fusing all the warped atlases into a single segmentation with a locally weighted approach (Sabuncu et al., 2010). We adjust the standard deviation of the likelihood model in the label fusion by testing several values for it (5 to 50 by increments of 5), and by keeping the one (30) yielding the best scores on the validation subset. The runtime for MAS was approximately one hour per case.

**Fig. 4:**
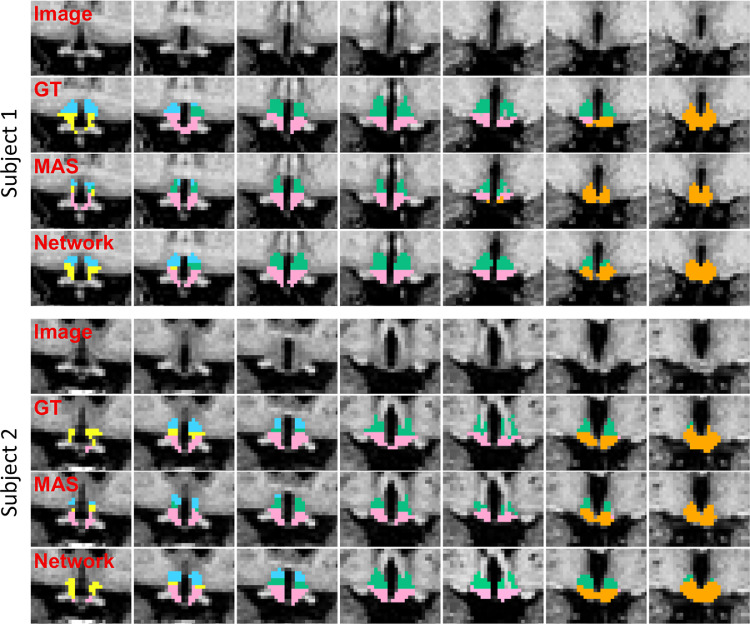
Comparison between coronal slices of manual and automated segmentations for two subjects randomly selected from the internal dataset. Slices are shown from anterior (left) to posterior (right). The four rows associated to each subject respectively illustrate the original image, the manual ground truth (GT), the segmentation produced by MAS, and the segmentation of the proposed network. Subunits colours follow the same scheme as in Fig. 3.

**Fig. 5:**
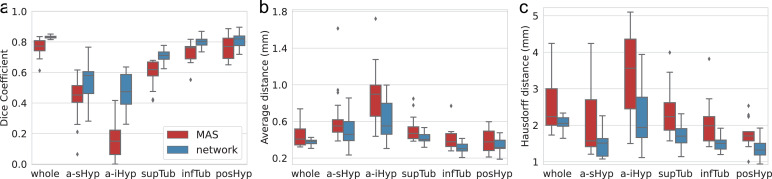
Comparison between MAS and our network on the test scans of the internal dataset: (a) Dice coefficients, (b) average boundary distance, and (c) Hausdorff distance. The improvement of the network is statistically significant for all metrics at the $10^{-3}$level (two-sided non-parametric Wilcoxon signed-rank tests) for the whole hypothalamus and all subunits. For each box, the central mark is the median; edges are the first and third quartiles; whiskers extend to 1.5 interquartile ranges around the median; and outliers are marked with ✦.

Visual inspection of the automated segmentations (Fig. 4) shows that the overall anatomy of the hypothalamic subunits is correctly learned by the network. The results obtained by the network for the three metrics, reported in Fig. 5, confirm this observation and exhibit the same tendency as for the intra and inter-rater variability experiments. Specifically, the whole hypothalamus yields a relatively high Dice coefficient of 0.83 as well as low values for average boundary distance (0.37 mm) and Hausdorff distance (2.04 mm). For the internal subunits, we observe that our method segments the posterior and tubular regions at the same level of accuracy as the whole hypothalamus. The much smaller anterior subregions obtain lower scores in terms of Dice, but are still competitive in terms of surface distance, e.g., the a-iHyp yields an average Hausdorff distance comparable to the whole hypothalamus. We emphasise that the assignment of a subject to either one of the training, testing, or validation subset has very little impact on these results (see Supplement 3).

In comparison, MAS yields a significantly less accurate segmentation of the hypothalamic subunits (Fig. 4), as it does not grasp the anatomy of the anterior nuclei, and exhibits smaller tuberal regions (both in axial and sagittal directions). Fig. 5 corroborates this visual assessment by showing that MAS is outperformed by the network for the whole hypothalamus and all subunits, according to all metrics. While these differences are all statistically significant at the $10^{-3}$level (two-sided non-parametric Wilcoxon signed-rank test), the biggest gap is observed for the a-iHyp, for which MAS obtains an average Dice scores below 0.2. Similarly, our model significantly outperforms MAS for the whole hypothalamus (0.07 difference in Dice scores, and 0.50 mm in Hausdorff distance). The performance difference between the two methods is more subtle in the tuberal and posterior units, for which the maximum gap in average boundary distance is 0.10 mm.

**Fig. 6:**
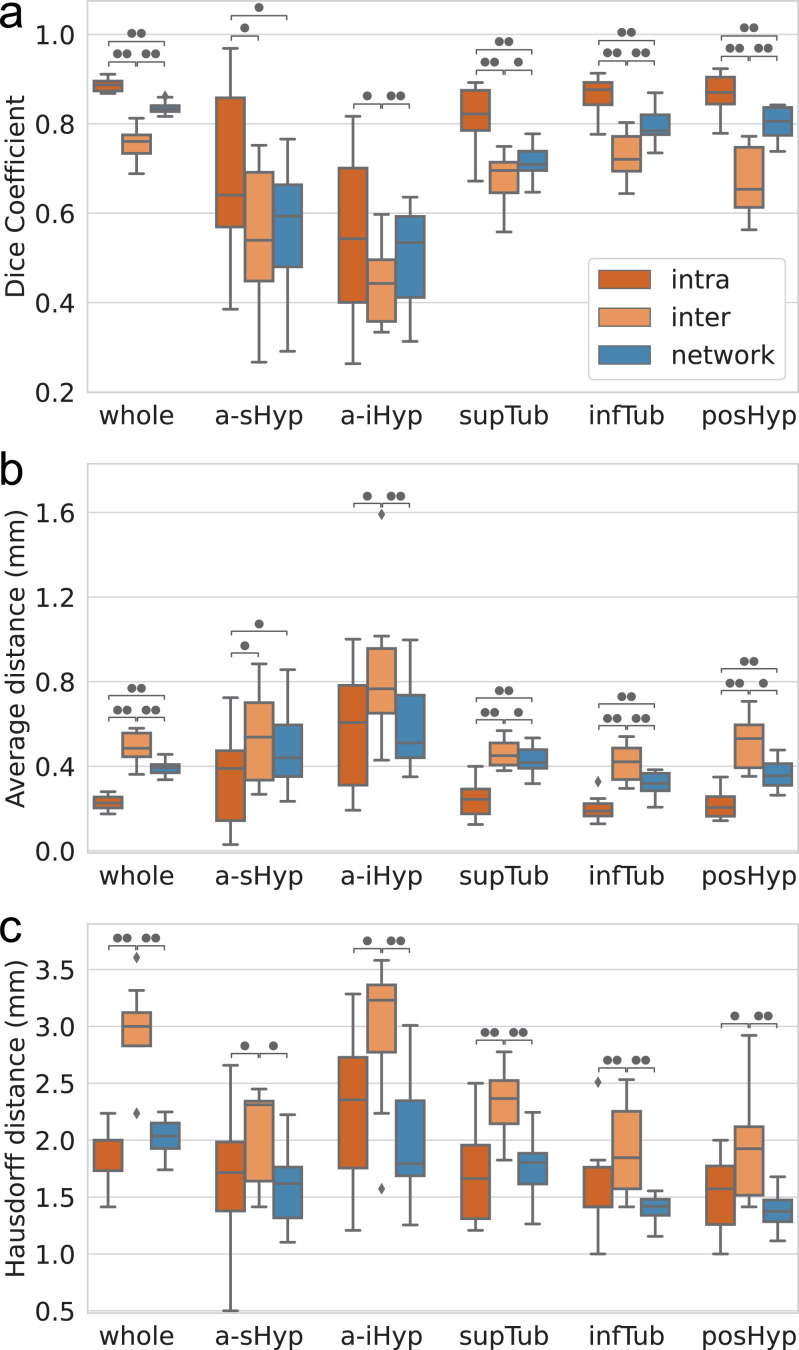
Comparison between the intra-rater, inter-rater, and automated segmentations scores on the ten subjects from the variability experiment: (a) Dice coefficients, (b) average boundary distance (mm), and (c) Hausdorff distance (mm). Statistical significance (two-sided non-parametric Wilcoxon signed-rank test) is represented by black circles (•*p* < 0.05, ••*p* < 0.01).

In order to put these results in context, particularly the structures with lower Dice, we compare the similarity scores of the automated segmentations with the scores obtained in the reproducibility experiments. This comparison is exclusively performed on the ten subjects of the first experiment, which are all part of the testing subset. Fig. 6 shows box plots for the three accuracy metrics as well as the statistical significance levels (two-sided non-parametric Wilcoxon rank-signed tests). Our model achieves better results than inter-rater reliabilities for the whole hypothalamus, and for almost all subregions (a-iHyp, supTub, infTub and posHyp), with p-values all lower than 0.01 (except for three cases where *p* < 0.05). The automated framework presents scores slightly better than the inter-rater accuracies for the a-sHyp unit, even if no significant difference can be inferred from the statistical tests, except for the Hausdorff distance of the a-iHyp subfield (p=0.013).

Since we use the segmentations of the expert rater as ground truth, the intra-rater similarity scores constitute the theoretical upper bound for the accuracy of the automated segmentation. Thus, it is not surprising that the intra-rater Dice coefficients and average distances are better than our method for the whole hypothalamus, as well as the tubular and posterior regions (Fig. 6(a,b)), with p-values all lower than 0.01. Nevertheless, the gap for these units is moderate considering their small size, since the difference between Table 4 reports Cohen’s d between control and AD populations the average Dice coefficients of the two never exceeds 0.10, which translates into a maximum average distance difference of only 0.17 mm. Moreover the difference between intra-rater and automated scores disappears for the Hausdorff distance (Fig. 6(c)), for which no significant difference can be inferred. The results obtained for the anterior region are even closer to the intra-variability level, especially for the a-iHyp unit, which yields the same average Dice score and presents similar distributions for the average and Hausdorff distances (Fig. 6).

#### 3.3.3 Robustness to differences in acquisition

A crucial aspect of the evaluation is testing the robustness of our approach using scans acquired on different hardware platforms and different T1-weighted sequences than the ones used in training. With this purpose, we use 675 scans from the highly heterogeneous ADNI dataset, which includes subjects spanning wide age range, some with severe AD-related atrophy, scanned with a variety of MR scanners using different sequences. Since manual delineations are not available for this dataset, we perform a visual QC analysis on the automated segmentations of these 675 scans. While visual assessment is not as informative as Dice scores computed against manual segmentations, it enables evaluation on a much larger sample, covering a much wider spectrum of variability in terms of anatomy and MR acquisition.

**Fig. 7:**
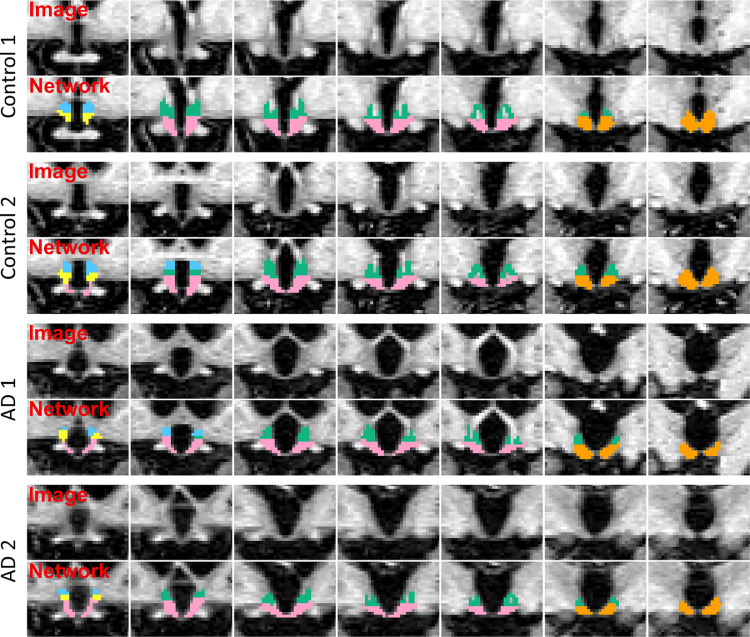
Coronal slices of segmentations produced by the network for four subjects randomly selected from the ADNI dataset. The first two cases are drawn among the control group, and the other two among AD subjects. Slices are shown from anterior (left) to posterior (right).

In this analysis, we first retrain the network with all the 37 manually labelled subjects. This new model, which we have made publicly available along with the code, is used to automatically segment the 675 scans. Then, the expert rater of the first experiment visually evaluates the quality of the segmentations produced by the network based on a pass/fail assessment. A segmentation is judged as a “pass”, when the expert believes it could robustly be used in an neuroimaging study involving the hypothalamic subunits. We emphasise that the QC is performed blindly of the age, gender, and medical condition (control, MCI, or AD) of the subjects. Despite the high variability in image acquisition (including head positioning) and anatomy (including atrophy patterns linked to normal ageing and AD), the network produces satisfying segmentations for 669 scans (see examples Fig. 7). QC only fails in six cases, therefore yielding a very low rejection rate of 0.89%. We identify two main reasons as probable causes for failure (Fig. S5): extreme set-up for head-positioning (four cases with rotation superior to 60^∘^ around right-left axis), and scans of poor quality (two cases).

**Fig. 8:**
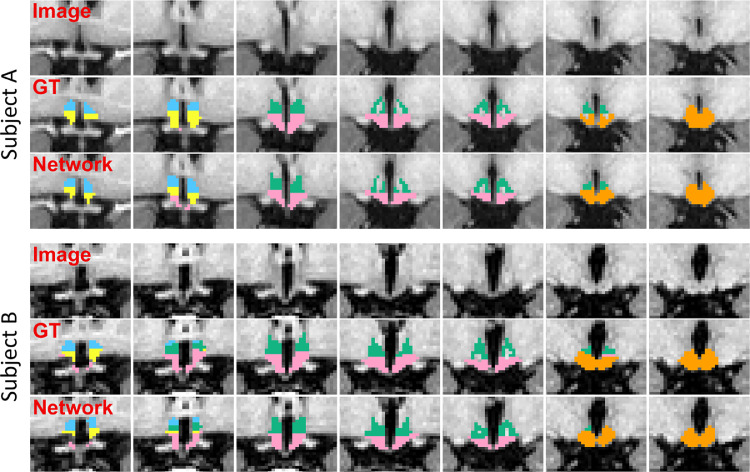
Coronal slices of segmentations produced by the network for two subjects of the external dataset (one from each subdataset).

In order to precisely quantify the robustness of this model, we test it on the external dataset of four scans, which were delineated by the expert rater. We evaluate the accuracy of the automated segmentations by computing the same three metrics as before. Even if the results are not directly comparable (due to differences in training and testing data), the scores (shown in Table 3) yielded by this model are very similar to the results obtained on the internal dataset, therefore demonstrating that the proposed model can robustly generalise to unseen datasets (see examples in Fig. 8).

**Table 3:** Average scores and associated standard deviations obtained by the proposed network on the external dataset.

	whole	a-sHyp	a-iHyp	supTub	infTub	posHyp
Dice coefficient	0.84 ± 0.01	0.57 ± 0.09	0.51 ± 0.12	0.67 ± 0.03	0.79 ± 0.06	0.79 ± 0.04
Average distance (mm)	0.42 ± 0.12	0.46 ± 0.11	0.54 ± 0.13	0.51 ± 0.14	0.31 ± 0.04	0.32 ± 0.08
Hausdorff distance (mm)	2.23 ± 0.70	1.70 ± 0.23	1.76 ± 0.28	2.28 ± 0.60	1.54 ± 0.23	1.38 ± 0.17

#### 3.3.4 Alzheimer’s disease volumetric study

In the fourth and final experiment, we assess the ability of the trained neural network to reliably segment MRI scans different from the training data, in the case of a neuroimaging group study, which represents the main application that we envision for this method. More specifically, we employ subjects from the ADNI dataset to indirectly evaluate the ability of the presented algorithm to detect atrophy patterns in AD (Callen et al., 2001; Ishii and Iadecola, 2015; Loskutova et al., 2010; Vercruysse et al., 2018).

In this experiment, we reuse the network from the previous experiment, i.e. the model trained on all 37 manually labelled scans, to run a volumetric study on the controls and AD subjects (317 subjects in total). Volumes are computed from the soft segmentations, i.e., the output of the softmax layer. This enables to account for segmentation uncertainties and, to some extent, for partial volume effect. All measured volumes are corrected for age and Intercranial Volume (ICV) using a general linear model. The ICVs are estimated with FreeSurfer (Fischl, 2012).

We analyse differences in the volumes of hypothalamic subunits between controls and diseased subjects using Cohen’s d and statistical significance tests. If *μ_C_*, $s_{C}^{2}$and *μ_A_*, $s_{A}^{2}$designate the means and variances of two volume populations of size *n_C_* and *n_A_*, where *C* stands for Controls and *A* for AD subjects, Cohen’s d is given by:

(6) $$d=\frac{\mu_{C}-\mu_{A}}{s},\;s=\sqrt{\frac{\left(n_{C}-1\right)s_{C}^{2}+\left(n_{A}-1\right)s_{A}^{2}}{n_{C}+n_{A}-2}}.$$

An effect size is considered small if its Cohen’s d is inferior to 0.2, and large if it is above 0.8 (Cohen, 1988). We also perform unpaired, one-tailed t-tests in order to test whether the observed volume differences are statistically significant.

Table 4 reports Cohen’s d between control and AD populations. Our automated algorithm detects large effect sizes of respectively 0.87 and 1.04 for the whole left and right hypothalamus, respectively. The network is able to detect similar, subunit-specific atrophies in anterior and posterior subunits (*d* ≥ 0.91). The disparity between both populations is largest for the a-sHyp and a-iHyp subregions, where the Cohen’s d exceeds 1 for all regions except the right a-iHyp. These large volume differences are statistically significant with very small p-values for the *t*-tests ($p<10^{-13}$).

**Table 4:** Cohen’s d measure between Control and AD populations for right and left whole hypothalami and subunits. *P*-values for one-tailed t-tests are shown in parentheses.

Side	whole	a-sHyp	a-iHyp	supTub	infTub	posHyp
Left	0.87 ($1\times 10^{-13}$)	1.28 ($3\times 10^{-25}$)	1.05 ($1\times 10^{-18}$)	0.57 ($4\times 10^{-7}$)	0.23 ($2\times 10^{-3}$)	0.91 ($1\times 10^{-14}$)
Right	1.04 ($4\times 10^{-18}$)	1.04 ($2\times 10^{-18}$)	0.92 ($6\times 10^{-14}$)	0.63 ($3\times 10^{-8}$)	0.44 ($7\times 10^{-5}$)	0.97 ($4\times 10^{-16}$)

Differences are lower in the tubular region, where the Cohen’s d values for the supTub and infTub range from 0.23 to 0.63. These results are still statistically significant with very small p-values ($10^{-3}<p<10^{-8}$), even if the differences are slightly smaller than for the anterior and posterior subunits.

## 4 Discussion

In this work, we have presented the first automated tool to segment the whole hypothalamus and its subnuclei. This task is challenging because of the lack of contrast in the hypothalamic region, which is mainly surrounded and composed by grey matter structures (Saper, 1990). This partly explains the less accurate results for the anterior subunits, where the boundary between a-iHyp and a-sHyp is faint as it is only defined by grey matter contrast. Moreover, cerebrospinal fluid and few white matter cell groups such as the fornix, the diagonal band of Broca, or the mamillo-thalamic tracts are also present in the hypothalamic region (Baroncini et al., 2012). In addition to being barely visible at 1 mm resolution, these white matter structures induce partial voluming effects, which hinder segmentation accuracy.

Despite the lack of contrast, the intra-rater setup exhibits a high level of reproducibility in terms of average boundary distance, especially for the posterior and tubular regions, which also yield low Hausdorff distances – comparable to those of the whole hypothalamus. However, the intra-rater Dice coefficients are lower than those usually reported for other whole brain structures (Fischl, 2012). This is explained by the small volumes of the hypothalamic subunits, especially for the anterior subregions, which also present overall flat and narrow shapes that negatively impact the Dice coefficients. The challenging nature of hypothalamic subunits segmentation is more apparent in the inter-rater variability, where the obtained scores are noticeably below the intra-rater level. It should be noted that our experiments involve two raters in total, including an expert and a junior rater. While having the former train the latter eliminates biases due to differences in labelling protocols, the junior rater may not be representative of a fully trained neuroanatomist. This may have affected the accuracy of the segmentations in the inter-rater study, especially for structures where boundary tracings rely on anatomical landmarks that may be hard to identify.

The second experiment suggests that, despite these challenges, our automated method is able to precisely learn the anatomy of the hypothalamus and its subunits. Indeed, this experiment demonstrates that the presented method: (*i*) segments the posterior and tubular subregions with the same level of precision as the whole hypothalamus, and (*ii*) significantly outperforms MAS (thoroughly validated and widely used in neuroimaging) for the whole hypothalamus and all subunits, while running orders of magnitude faster at test time. In comparison with a recent deep learning approach for whole hypothalamus segmentation (Rodrigues et al., 2020), our model shows an improvement of 0.07 in Dice coefficient. Although these results are not directly comparable due to differences in datasets, the improvement may be because of our more aggressive data augmentation scheme, including: linear and elastic transformations, bias field corruption, and intensity augmentation. No comparison with other automated methods is possible for the hypothalamic subunits, as this work is the first to automatically achieve such segmentation.

The proposed network is also demonstrated to significantly surpass inter-rater precision level for tubular and posterior subunits, as well as for the a-iHyp unit. By accurately learning the labelling patterns of the expert rater, the network makes better decisions for peripheral voxels than the second rater. The fact that no difference can be inferred from the statistical tests for the a-sHyp subregion suggests that, despite lower accuracy scores (due to the small size and lack of contrast), it can still be segmented at inter-rater precision level.

Because the intra-rater study constitutes the upper bound in terms of segmentation accuracy, it was also expected that its scores would be significantly superior to the ones of the proposed framework. Nevertheless, the gap between the two for Dice scores and average boundary distance is mild, especially for the particularly difficult anterior regions, whereas no distinction can be detected for the Hausdorff distance. This very encouraging result indicates that the network has correctly learned the overall structure shapes and does not commit bigger mistakes than the human expert. We emphasise that manual segmentations rely on T1 and T2-weighted scans previously registered to a standard template, whereas our method only uses T1-weighted brain scans that are not preprocessed in any way. This choice was motivated by the fact that we designed this tool to be publicly available and widely applicable, and thus to require the least possible number of MR contrasts.

In the third experiment, we retrain our framework on all the available scans, and we evaluate the obtained model by testing it on a validation dataset comprising four scans with ground truth delineations. We complement this robustness study with direct visual assessment of the segmentations produced by the network for the heterogeneous ADNI dataset. The high scores obtained by the retrained model combined with the low rejection rate (below 1%) of the quality control analysis, demonstrate that our method is robust to high variability in T1-weighted scans.

Finally, we validate our approach indirectly by quantifying the effect sizes between subunit volumes of control and diseased subjects in a population study. This experiment represents a typical scenario in which our tool will be used. The most significant volume differences are detected in both anterior and posterior regions. Anterior nuclei have already been reported to undergo severe atrophy in AD (Baron et al., 2001) especially in the suprachiasmatic nucleus (responsible for regulation of the circadian cycle) (Baloyannis, Mavroudis, Mitilineos, Baloyannis, Costa, 2015, Harper, Stopa, Kuo-Leblanc, McKee, Asayama, Volicer, Kowall, Satlin, 2008, Swaab, Fliers, Partiman, 1985), parts of the supraoptic nucleus (involved in ageing mechanisms) (Baloyannis, Mavroudis, Mitilineos, Baloyannis, Costa, 2015, Goudsmit, Hofman, Fliers, Swaab, 1990), and the paraventricular nucleus (implicated in satiety perception). Even if the anterior subunits are the less accurately segmented, this experiment shows that our method is precise enough to detect subtle volume changes in these regions. The results for the posterior region atrophy are also in agreement with previous studies (Callen, Black, Caldwell, Grady, 2004, Copenhaver, Rabin, Saykin, Roth, Wishart, Flashman, Santulli, McHugh, Mamourian, 2006, Fronczek, van Geest, Frölich, Overeem, Roelandse, Lammers, Swaab, 2012, Nestor, Fryer, Smielewski, Hodges, 2003). This can be explained by the fact that this region is mainly constituted by the mamillary bodies, which are connected via the fornix to the hippocampus (known to be severely affected by AD (Fox et al., 1996), and by the lateral hypothalamus, which holds roles in ageing, appetite and sleeping cycles (McDuff and Sumi, 1985).

The atrophy of the tubular subunits in AD has been less frequently described in the literature and has been found to be smaller than for the other subregions (Saper and German, 1987). This finding is in agreement with the lower effect sizes obtained for both infTub and supTub units. A significant difference is still found in the supTub region ($p<10^{-6}$), which contains parts of the paraventricular, lateral and dorsomedial (associated with ageing functions) nuclei. Nevertheless this distinction is less clear for the infTub region, which is responsible for functions less associated with AD (metabolic and hormonal signalling, sexual behaviour (Bao, Swaab, 2011, Goudsmit, Hofman, Fliers, Swaab, 1990)). Even if the tubular subunits accounts for two thirds of the total hypothalamic volume, our algorithm still detects strong effect sizes for the whole hypothalamus, which are comparable to that of the most affected anterior subunits (Cohen’s d values of 0.87 and 1.04 for respectively left and right hypothalami, $p<10^{-12}$). Overall, the coherence of these volumetric measurements further indicates that our method is robust to high variability in T1-weighted scans, including pathologies deeply affecting the structure of the hypothalamus.

More generally, our aggressive data augmentation strategy is found to greatly increase the robustness of the proposed model, as highlighted by the poor scores obtained when ablating augmentation. This observation is in agreement with recent publications, which show that aggressive data augmentation (even beyond realistic limits) increases generalisation at testing (Billot, Greve, Van Leemput, Fischl, Iglesias, Dalca, Chaitanya, Karani, Baumgartner, Becker, Donati, Konukoglu, 2019, Zhao, Balakrishnan, Durand, Guttag, Dalca, 2019). We believe this partly explains the ability of the network to successfully generalise to the heterogeneous ADNI dataset, which includes scans with intensity profiles that are very different from those of the training data. Moreover, the adaptability of the proposed method is further demonstrated in the cross-validation studies, where our framework is shown to generalise well to populations with different characteristics from the training subjects (Supplement 3). However, the accuracy of the produced segmentations could be limited by some forms of variability, such as extreme head-positioning, scans of bad quality, or lesions, which are not currently modelled. Moreover, our model is trained on 1 mm resolution scans and is thus unable to capitalise on higher resolutions. While the vast majority of data in neuroimaging has 1 mm voxel size, new labelled datasets will be required to train networks that exploit the higher resolutions that are becoming increasingly available (particularly at 7T), as well as contrasts other than T1.

## 5 Conclusion

In this paper, we have presented a tool to automatically segment the hypothalamus and its associated subregions in MR T1-weighted brain scans. The proposed framework does not require any preprocessing and is based on the use of a convolutional network, permitting extremely fast structure segmentation at inference (less than a second on a GPU, around ten seconds on a standard modern CPU). The algorithm is completed by an aggressive data augmentation model, which enables accurate and robust hypothalamic segmentation of scans from widely different sources. In a first set of experiments, we employed a dataset of 37 subjects to compare our approach against a MAS baseline, and manual delineations. We demonstrated that our automated tool consistently exceeds MAS as well as human inter-rater accuracy level, and nearly reaches intra-rater precision. We further validated the accuracy and robustness of the proposed method by first showing that it maintained its high accuracy performances on an external dataset of four scans, and then with a quality control analysis performed on a broader subset of 675 heterogeneous scans from the multi-site ADNI dataset, which yielded a rejection rate below 1%. Finally, we evaluated our approach by applying it to a volumetric analysis on 317 ADNI scans, which closely represents the type of application the method is designed for. Using the automated measurements, we managed to accurately replicate neuropathological atrophy findings associated with AD, by detecting significant volume differences between controls and AD subjects in specific subunits.

Future work will focus on extending this framework to other MRI modalities. Moreover, we believe that the quality of the automated segmentations could be increased by exploiting additional MRI contrasts such as T2-weighted scans, which are already used in the manual delineation protocol. Another possible line of work could aim at building models operating on MRI scans of higher resolution, in order to segment the hypothalamic subunits with even higher precision.

This publicly available automated tool will enable researchers around the world to conduct studies of the hypothalamus and its subunits *in vivo*, in a reproducible manner, and at a large scale. Therefore, our open-source tool has the potential to help unravel the involvement of the hypothalamus in a number of vital functions as well as neurodegenerative diseases like AD, Parkinson’s Disease or frontotemporal dementia, which represent a huge burden on society.

## CRediT authorship contribution statement

**Benjamin Billot:** Data curation, Formal analysis, Investigation, Methodology, Software, Validation, Visualization, Writing - original draft, Writing - review & editing. **Martina Bocchetta:** Conceptualization, Data curation, Investigation, Methodology, Resources, Supervision, Validation, Writing - review & editing. **Emily Todd:** Data curation, Investigation, Resources, Validation, Writing - review & editing. **Adrian V. Dalca:** Conceptualization, Methodology, Software, Supervision, Writing - review & editing. **Jonathan D. Rohrer:** Conceptualization, Funding acquisition, Resources, Supervision, Writing - review & editing. **Juan Eugenio Iglesias:** Conceptualization, Data curation, Funding acquisition, Methodology, Project administration, Software, Supervision, Writing - review & editing.

## Appendix A Supplementary materials

Supplementary Data S1 Supplementary Raw Research Data. This is open data under the CC BY license http://creativecommons.org/licenses/by/4.0/ Supplementary Data S1
