# Supplementary material for: Automated segmentation of the hypothalamus and associated subunits in brain MRI
Source: Neuroimage. 2020 Dec;223:117287. doi: 10.1016/j.neuroimage.2020.117287 (PMC8417769; doi:10.1016/j.neuroimage.2020.117287)
Supplement: Supplementary Data S1 — Supplementary Raw Research Data. This is open data under the CC BY license http://creativecommons.org/licenses/by/4.0/ [file mmc1.pdf]

Automated segmentation of the Hypothalamus and associated subunits in brain MRI, Supplementary materials

Billot, Bocchetta, Todd, Dalca, Rohrer, Iglesias

Supplement 1: Augmentation examples

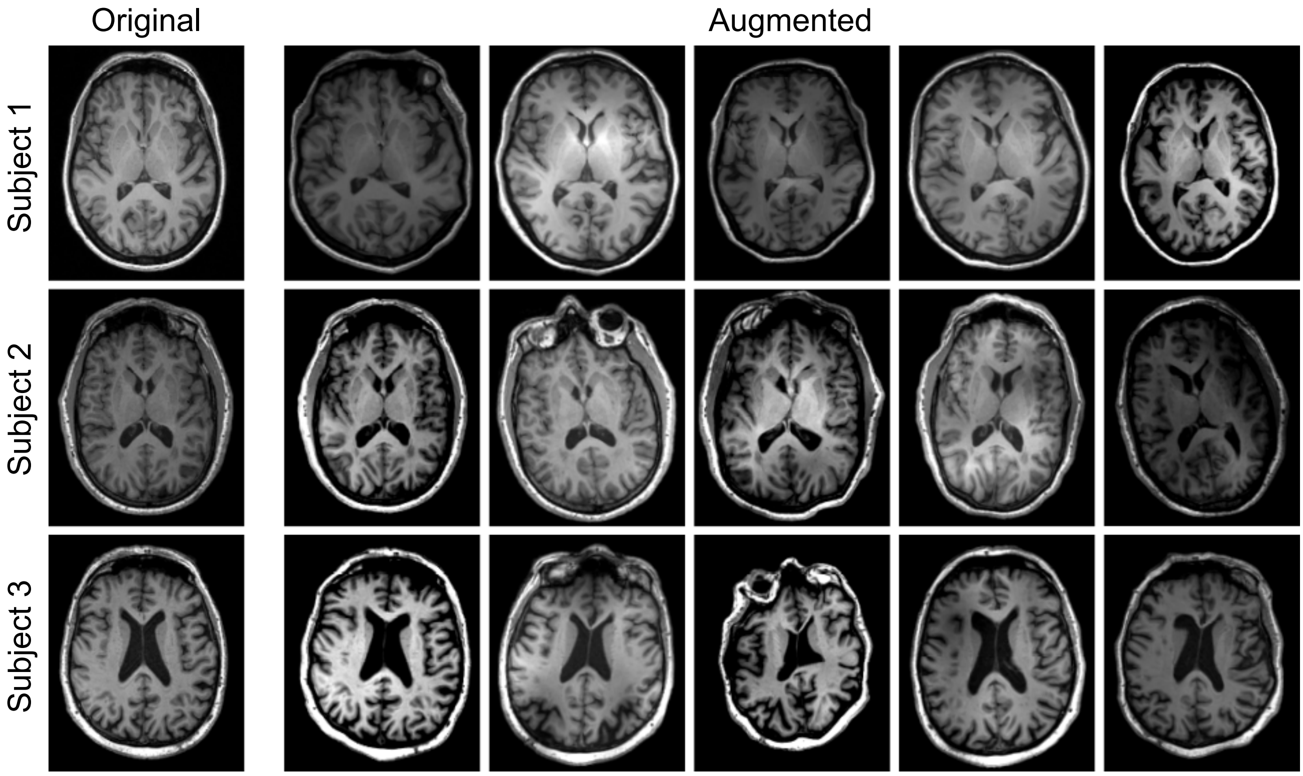

Figure S1: Axial slices of additional training scans obtained with the proposed augmentation model. We apply a wide spectrum of positionings, spatial deformations, noise levels, contrast variations and bias fields. Original images are shown on the left, with examples of random augmentations represented in subsequent columns. Each row corresponds to a different subject. The displayed slices correspond to approximately the same spatial location.

Supplement 2: Learning curves

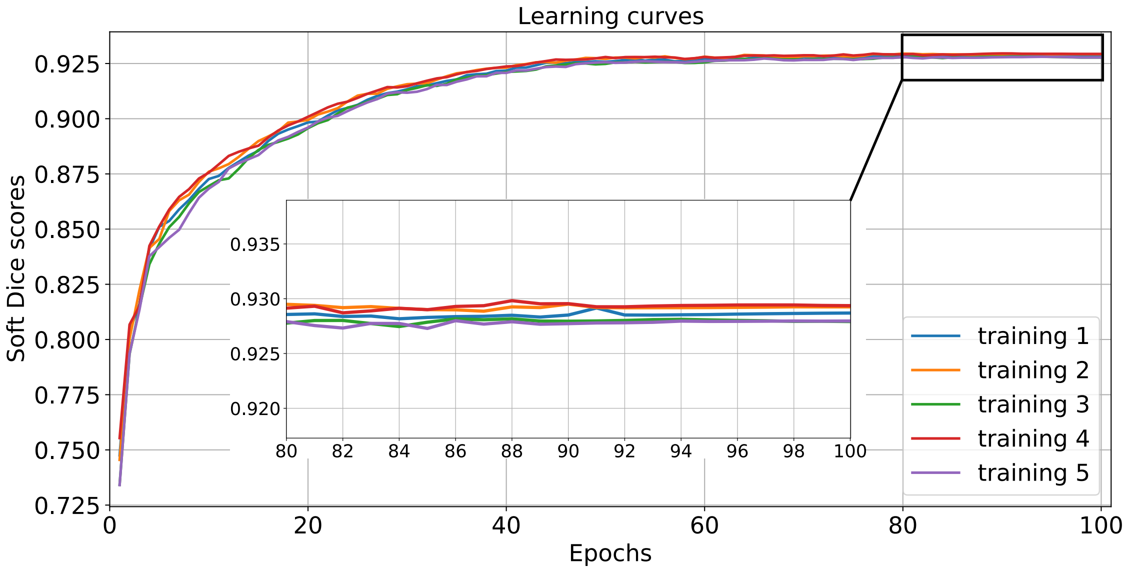

Figure S2: Learning curves obtained by the optimal architecture for each training run. The optimal architecture is trained five times to reduce the stochastic fluctuations across runs. The embedded graph is a zoom on the learning curves for the last 20 epochs.

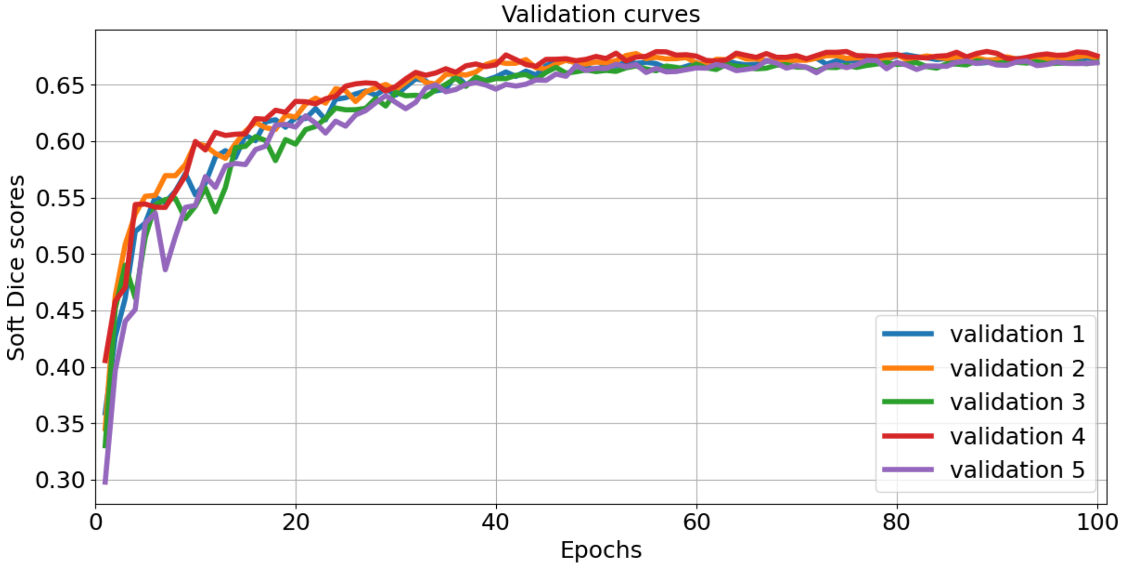

Figure S3: Validation curves obtained with the optimal architecture for the five training runs. These curves show that the network did not overfit the training data.

Supplement 3: Cross-validation studies and robustness to population types

This experiment aims at evaluating the adaptability of the proposed framework when tested on a population with different characteristics from the training subjects. With this purpose, we run three separate 2-folds cross-validation studies with different subject-assignment strategies: first, random assignment to the two folds; second, separating the youngest subjects from the oldest; and third, splitting into healthy controls and subjects suffering from frontotemporal dementia (FTD). We separately train a model on each fold and evaluate its accuracy on the scans of the corresponding opposite fold. Figure S4 shows that the Dice coefficients obtained in the young vs. old, and controls vs. FTD set-ups are very similar to the scores yielded by the networks trained on the random folds, thus highlighting the ability of our method to generalise well to different population types.

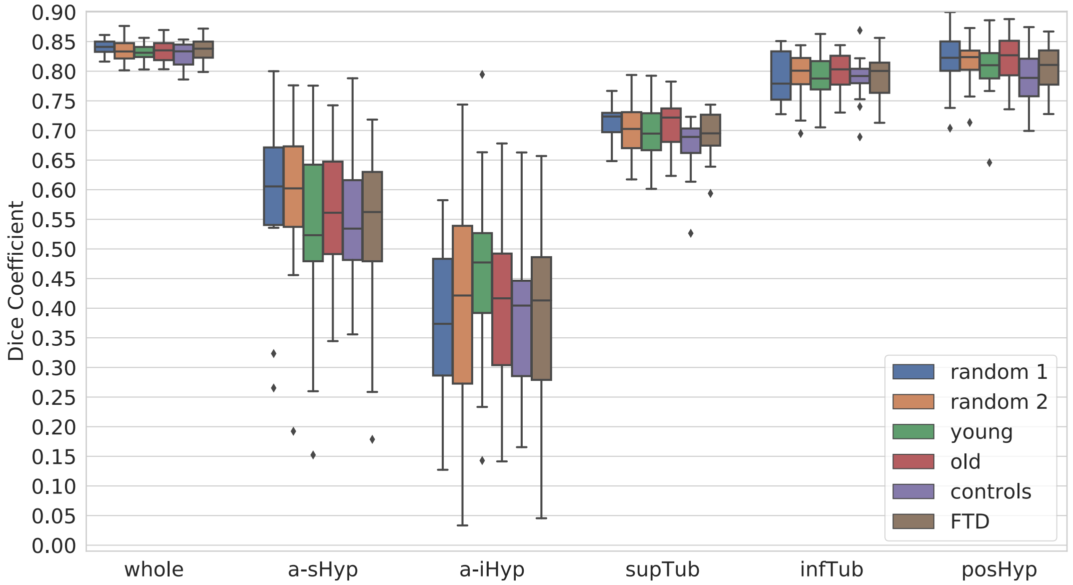

Figure S4: Dice scores obtained in three cross-validation studies with different subject-assignment strategies: random splitting, young vs. old, and controls vs. frontotemporal dementia (FTD). Each box presents the results obtained on a population by the network trained on the corresponding opposite fold.

Supplement 4: Causes of failure in the Quality Control analysis

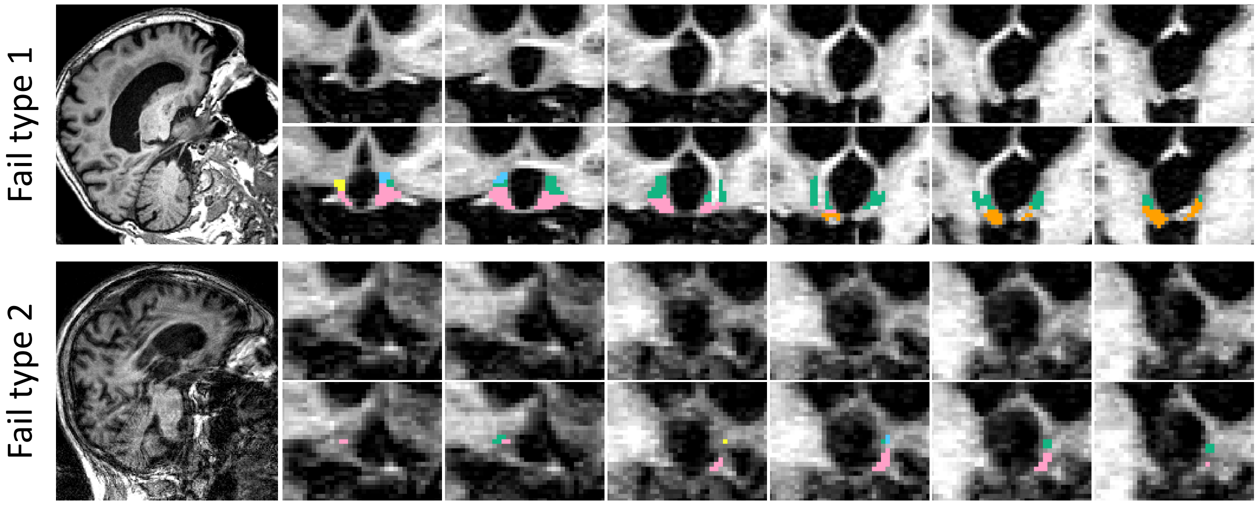

Figure S5: Coronal slices of subjects representative of the two types of failure identified in the QC analysis. The first source of failure stems from extreme head positioning (rotation of more than 60° relatively to the horizontal axis), which goes beyond the linear deformation of the augmentation model. We see that this setting particularly affects the segmentation of the posterior region, leaving the anterior regions relatively spared. The second cause of failure simply stems from abnormally low signal-to-noise ratios.
